# Lymphoepithelial Cyst of the Hypopharynx

**DOI:** 10.1155/DTE.2.53

**Published:** 1995

**Authors:** Masahiro Kawaida, Hiroyuki Fukuda, Akihiro Shiotani, Naoyuki Kohno

**Affiliations:** 1 Department of Otolaryngology Tokyo Metropolitan Ohtsuka Hospital Tokyo, Japan, 2-8-1, Minamiohtsuka Toshima-ku Tokyo 170 Japan; 2 Department of Otolaryngology Keio University School of Medicine Tokyo, Japan, 35 Shinanomachi Shinjuku-ku Tokyo 160 Japan; 3 Department of Otolaryngology Juntendo University School of Medicine Tokyo, Japan, 2-1-1, Hongo Bunkyo-ku Tokyo 113 Japan

## Abstract

A rare case of lymphoepithelial cyst formed in the piriform sinus of the hypopharynx is reported. Histopathological examination revealed a lymphoepithelial cyst. It was removed by laryngomicrosurgical technique using a side-opened direct laryngoscope. Because this cyst was wide-based on the antero-medial region in the right piriform sinus of the hypopharynx, the mucous membrane around the cyst was incised electrosurgically and then detached to facilitate removal. In this paper, we describe our surgical procedure for removing the cyst in this case and discuss the possible causes of the disease.


*Diagnostic and Therapeutic Endoscopy*, 1995, Vol. 2, pp. 53–56
Reprints available directly from the publisher
Photocopying permitted by license only

© 1995 Harwood Academic Publishers GmbH
Printed in Singapore

### Lymphoepithelial Cyst of the Hypopharynx: A Case Report

MASAHIRO KAWAIDA,* HIROYUKI FUKUDA,** AKIHIRO SHIOTANI** and NAOYUKI KOHNO***

**Department of Otolaryngology, Tokyo Metropolitan Ohtsuka Hospital, Tokyo, Japan, 2-8-1, Minamiohtsuka, Toshima-ku, Tokyo 170, Japan; **Department of Otolaryngology, Keio University School of Medicine, Tokyo, Japan, 35 Shinanomachi, Shinjuku-ku, Tokyo 160, Japan; ***Department of Otolaryngology, Juntendo University School of Medicine, Tokyo, Japan, 2-1-1, Hongo, Bunkyo-ku, Tokyo 113, Japan*

*(Received April 15, 1994; in final form March, 1995)*

A rare case of lymphoepithelial cyst formed in the piriform sinus of the hypopharynx is reported. Histopathological examination revealed a lymphoepithelial cyst. It was removed by laryngomicrosurgical technique using a side-opened direct laryngoscope. Because this cyst was wide-based on the antero-medial region in the right piriform sinus of the hypopharynx, the mucous membrane around the cyst was incised electrosurgically and then detached to facilitate removal. In this paper, we describe our surgical procedure for removing the cyst in this case and discuss the possible causes of the disease.

**KEY WORDS:** lymphoepithelial cyst, hypopharynx, side-opened direct laryngoscope, branchial cyst, laryngomicrosurgery

### INTRODUCTION

It is rare for a cyst to form in the hypopharynx, although some such cases have been reported (1–4). A hypopharyngeal lymphoepithelial cyst is especially rare. Removal of a large cyst from this area by the oral approach can be difficult. Thus, it is preferable to remove such a cyst by endopharyngeal microsurgery when it is still small. Previously, we described a side-opened direct laryngoscope developed to facilitate bimanual manipulation (5). We also reported a method for removing epiglottic cysts and hypopharyngeal retention cysts using this type of direct laryngoscope (6,7). Here, we performed microsurgery on a patient with a lymphoepithelial cyst in the hypopharynx using this method and the surgical procedure is described below.

### CASE REPORT

### Clinical Course

In December 1991, a 50-year-old man noted an abnormal feeling in his throat. This symptom did not resolve and he presented to our clinic on February 22, 1993. A flexible laryngofiberscopic examination revealed a pale yellow hemispheric cystic lesion arising from the antero-medial region of his left piriform sinus in the hypopharynx (Fig. 1a). The patient was admitted to our hospital for treatment on June 21, 1993.

On June 22, the lesion was removed using a side-opened direct laryngoscope, an electrosurgical instrument and a snare as described below. The hypopharyngeal cystic lesion was approximately 1 *cm* in diameter, and appeared as a hemispheric bulge on the antero-medial region of the right piriform sinus (Fig. 2a). Local fingings 12 weeks postoperatively revealed complete disappearance of the lesion (Fig. 1b). There has been no recurrence.

### Surgical Procedure

Laryngomicrosurgery was performed using a side-opened direct laryngoscope (Nagashima Medical Instruments Co., Ltd.) that facilitated bimanual manipulation (5–7). A laryngomicrosurgical snare and the longest electrosurgical instrument electrode presently available were inserted through the right side-opening of the direct laryngoscope (7). The surgery was performed under inhalation anesthesia by endotracheal intubation. The anterior tip of the side-opened direct laryngoscope was introduced into the hypopharynx. When the cyst appeared within view, the direct laryngoscope was self-retained with a holder and

Address for correspondence: Dr. Masahiro Kawaida, Department of Otolaryngology, Tokyo Metropolitan Ohtsuka Hospital, 2-8-1, Minamiohtsuka, Toshima-ku, Tokyo 170, Japan.

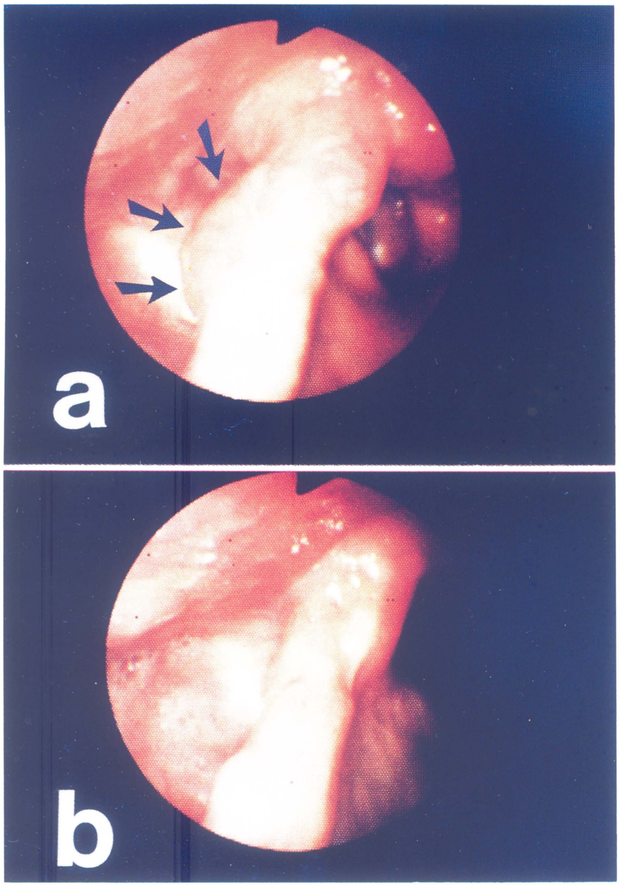

**Figure 1** Flexible laryngofiberscopic findings prior to, and following the surgical procedure. a (Preoperative), A hemispheric cystic lesion can be observed in the antero-medial region of the right piriform sinus. b (Postoperative), Complete disappearance of the cyst.

chest support. The mucous membrane near the base of the cyst was incised with an electrosurgical instrument and detached using forceps to expose the cyst wall (Fig. 2b, c). The base of the lesion was then snared and excised (Fig. 2d, 3). The entire surgical procedure was performed under microscopic observation.

### Histopathological Findings

The cyst demonstrated an outer squamous epithelial lining and the cystic cavity was covered by stratified squamous epithelium. There was abundant lymphoid tissue with germinal centers beneath the epithelium. Histopathologically, the lesion was identified as a lymphoepithlial cyst (Fig. 4).

### DISCUSSION

It is very rare for a cyst to form in the hypopharynx, although foregut cysts, branchial cysts and retention cysts of the hypopharynx occasionally have been reported (1,3,4). We have also reported two cases of hypopharyngeal retention cysts (7).

Microscopically, the cystic cavity was covered by stratified squamous epithelium, and lymphoid tissue with germinal centers was abundant beneath the inner epithelium. This lesion was identified histopathologically as a lymphoepithelial cyst that had formed in the antero-medial region of the piriform sinus.

Recently, it has been thought that lymphoepithelial cysts are the same as branchial cysts. Immunohistochemical studies have revealed similarities between the epithelial elements of branchial cysts and palatine tonsils and between the lymphoid tissue of branchial cysts and lymph nodes (8,9). Lymphoepithelial cysts (branchial cysts) are thought to arise from the cystic degeneration of epithelium enclosed with lymph nodes and tonsilar tissue (10). In our case, the hypopharyngeal cyst was formed in a region close to the lingual tonsil. Physical stimulation during the deglutition of food may have also led to the development of a lymphoepithelial cyst.

The treatment of hypopharyngeal cysts is size-dependent. The surgical removal of a small cyst is simple, and many of these cysts may be removed by endopharyngeal microsurgery using a direct laryngoscope. In the case of a very large cyst, dysphagia and dyspnea may occur. A tracheostomy is necessary if inhalation anesthesia by standard endotracheal intubation cannot be performed. It may be necessary to puncture and drain a large cyst to reduce its size prior to removal by an oral approach. In some cases, giant obstructing cysts may require a laryngofissure or lateral pharyngotomy for complete resection.

Previously, we have reported a method employing a side-opened direct laryngoscope, an electrosurgical instrument and a snare for the removal of hypopharyngeal retention cysts (7). This patient with a hypopharyngeal lymphoepithelial cyst was treated in a similar manner. It is important to control bleeding when removing a cyst using a direct laryngoscope. Profuse bleeding will occlude the view and interfere with the complete removal of the cyst. Electrocoagulation is recommended when hemostasis cannot be readily achieved.

The postoperative course of this patient has been satisfactory with no complications or recurrence.

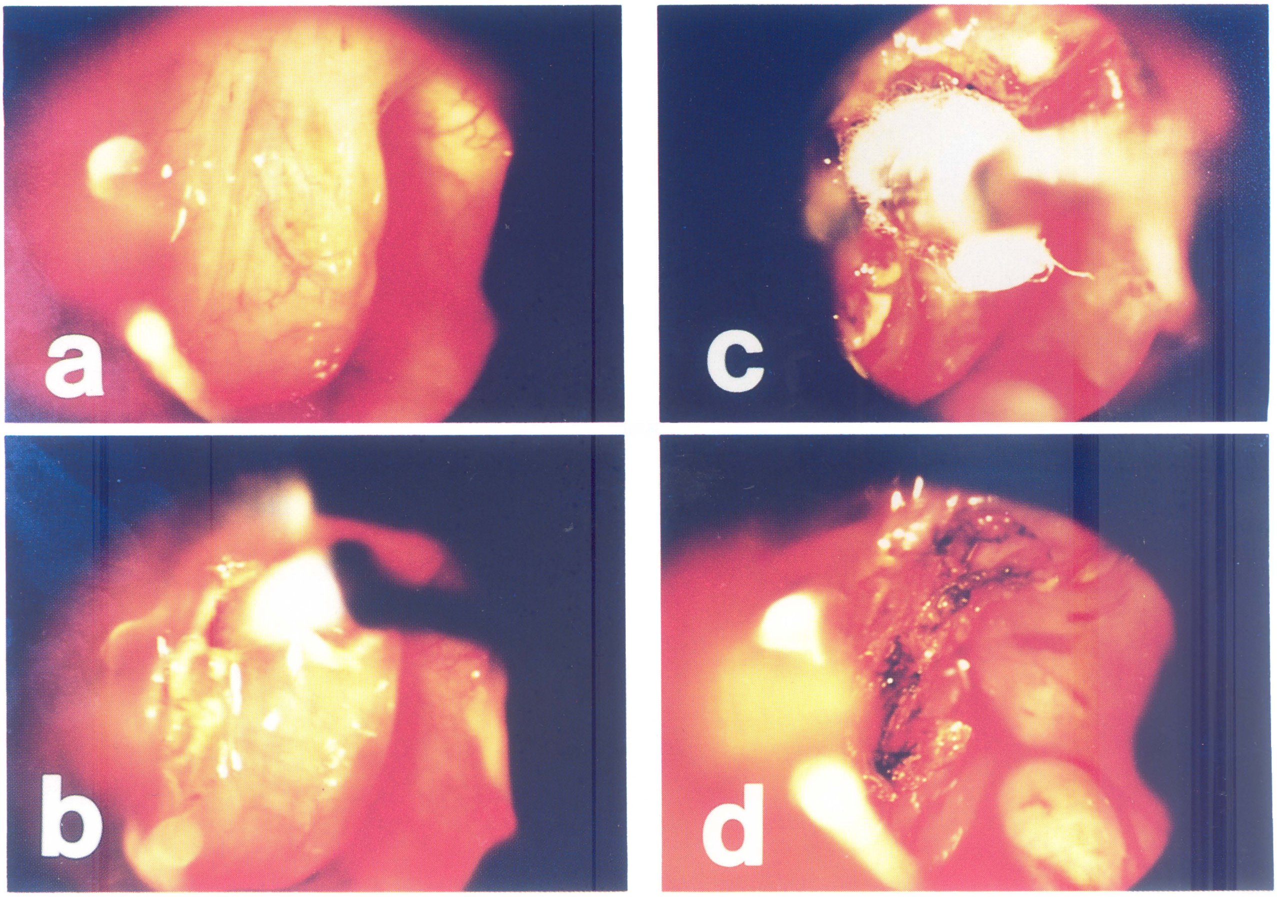

**Figure 2** Surgical findings. a, A wide-based cystic lesion is visible in the antero-medial region of the right piriform sinus. b, View of the mucous membrane around the cyst following incision with an electrosurgical instrument. c, View of the region around the cyst following detachment with a cotton swab held by forceps. d, Local findings at the operative site following resection of the cyst.

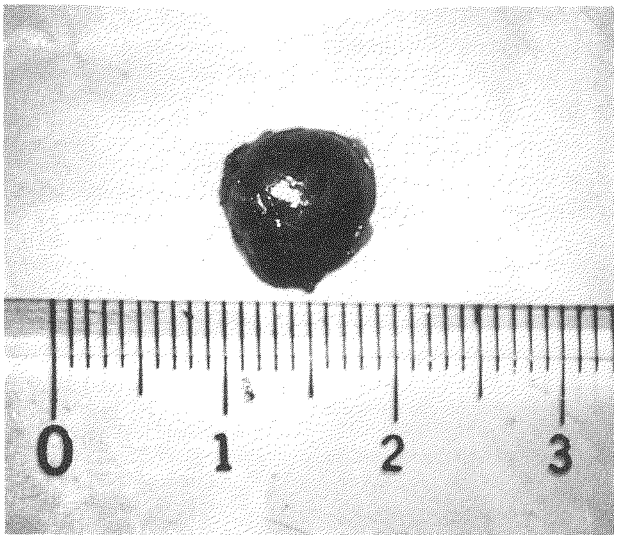

**Figure 3** Resected hypopharyngeal cystic lesion.

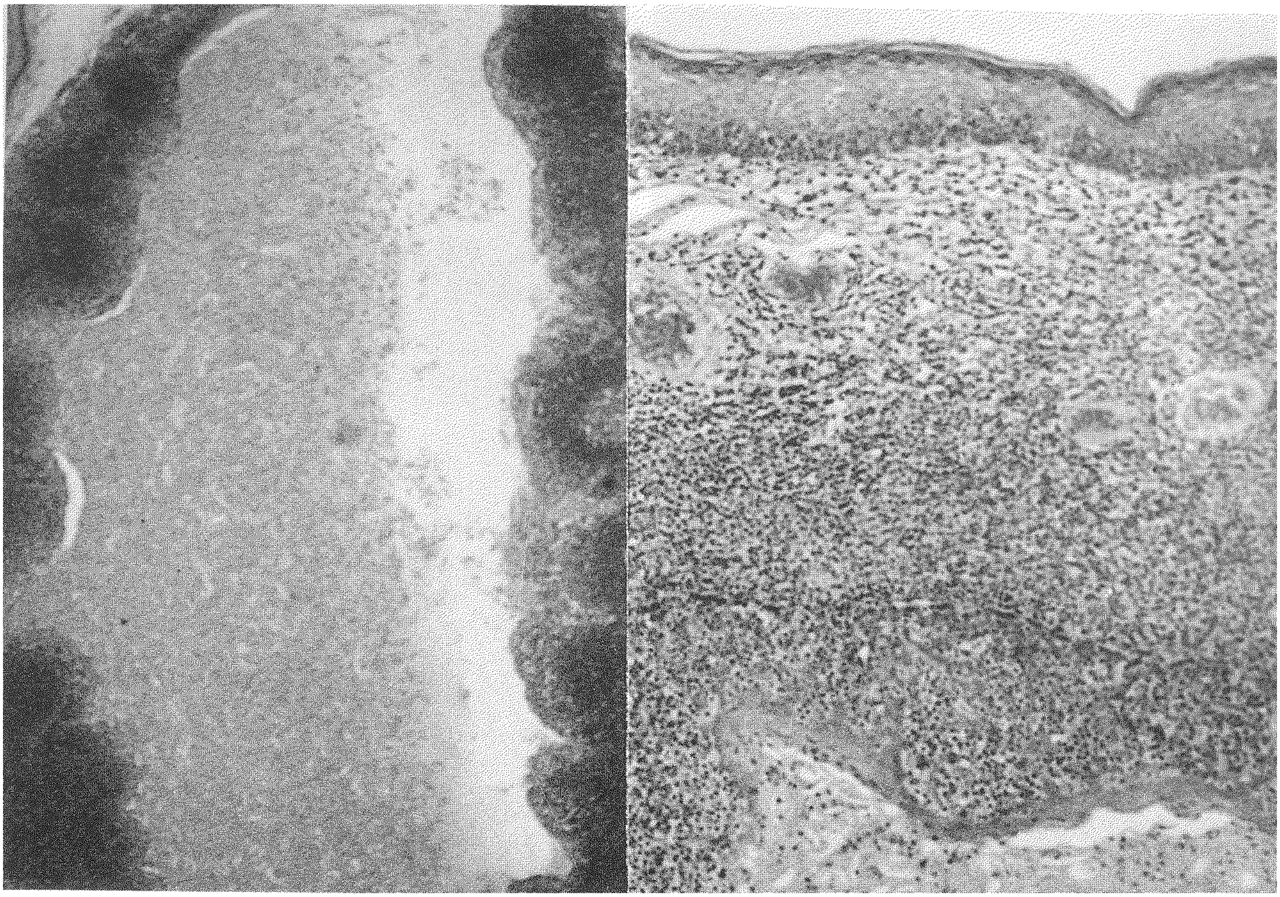

**Figure 4** Histopathological findings. Stratified squamous epithelium covering the cystic cavity and abundant lymphoid tissue with germinal centers beneath the epithelium (H.E. × 40, × 100).

### SUMMARY

We reported the case of a patient with a lymphoepithelial cyst which formed in the piriform sinus of his hypopharynx. A laryngomicrosurgical technique was performed using a side-opened direct larygoscope, an electrosurgical instrument and a snare, and the cyst was successfully removed. We discussed the clinical picture and histopathological findings in this case.

### REFERENCES

1. Canty TG, Henderson WH. Upper airway obstruction from foregut cysts of the hypopharynx. J Pediatr Surg 1975;10:807–812.
2. Kasliwal KC, Narayanan TK, Sohail MA. Pharyngeal cysts. J Indian Med Assoc 1977;69:158–160.
3. Boysen ME, De Besche A, Djupesland G, et al. Internal cysts and fistulae of branchaial origin. J Laryngol Otol 1979;93:533–539.
4. Ailhara Y, Egami H, Kobayashi Y, et al. Hypopharyngeal cyst; A case report. Pract Otol 1993;86:553–557.
5. Kawaida M, Fukuda H, Kano S, et al. Laryngomicrosurgery by use of a side-opened direct laryngoscope. In: Inouye T, Fukuda H, Sato T, Hinohara T, (eds.). Recent Advances in Bronchoesophagology, pp. 355–356, Elsevier Science Publishers B.V., Amsterdam, 1990.
6. Kawaida M, Kohno N, Kawasaki Y, et al. Surgical treatment of large epiglottic cysts with a side-opened direct laryngoscope and snare. Auris · Nasus · Larynx (Tokyo), 1992;19:45–50.
7. Kawaida M, Fukuda H, Shiotani A, et al. Surgical treatment for hypopharyngeal cysts with a side-opened direct laryngoscope. Auris · Nasus · Larynx (Tokyo), 1994;21:38–43.
8. Crocker J, Jenkins R. An immunohistochemical study of branchial cysts. J Clin Pathol 1985;38:784–790.
9. Wild G, Mischke D, Lobeck H, et al. The lateral cyst of the neck; Congenital or acquired? Acta Otolaryngol (Stockh), 1987;103:546–550.
10. Gorlin RJ. Face, lips, tongue, teeth, oral soft tissues, jaws, salivary glands and neck. In: Kissane JM (eds.): Anderson's Pathology, Eighth Edition. pp. 1002–1054, The C.V. Mosby Company, St. Louis, 1985.

